# 

**Published:** 1886-10

**Authors:** 


OCTOBER, 1886

### THE AMERICAN JOURNAL OF DENTAL SCIENCE.

EDITED BY

F. J. S. GORGAS, M. D., D. D. S.

PUBLISHED MONTHLY, $2.50 PER ANNUM.

VOL. 20 THIRD SERIES. NO. 6.

BALTIMORE:

SNOWDEN & COWMAN.

LONDON:
TRUBNER & CO., 60 PATERNOSTER ROW.

